# Antimicrobial stewardship: a definition with a One Health perspective

**DOI:** 10.1038/s44259-024-00031-w

**Published:** 2024-05-02

**Authors:** Rebecca Hibbard, Marc Mendelson, Stephen W. Page, Jorge Pinto Ferreira, Céline Pulcini, Mathilde C. Paul, Céline Faverjon

**Affiliations:** 1 IHAP, Université de Toulouse, INRAE, ENVT, Toulouse, France; 2 Division of Infectious Diseases & HIV Medicine, Department of Medicine, Groote Schuur Hospital, University of Cape Town, Cape Town, South Africa; 3 Advanced Veterinary Therapeutics, Newtown, NSW 2042 Australia; 4 Food and Agriculture Organization of the United Nations (FAO), Viale delle Terme di Caracalla, 00153 Rome, Italy; 5 Université de Lorraine, Inserm, INSPIIRE, Nancy, France and Université de Lorraine, CHRU-Nancy, Centre régional en antibiothérapie du Grand Est AntibioEst, Nancy, France; 6 Ausvet Europe, Lyon, France

**Keywords:** Epidemiology, Epidemiology

## Abstract

Antimicrobial stewardship (AMS) is a commonly advocated approach to address antimicrobial resistance. However, AMS is often defined in different ways depending on where it is applied, such that a range of definitions is now in use. These definitions may be functional and well-structured for a given context but are often ill-adapted for collaborative work, creating difficulties for intersectoral communication on AMS and complicating the design, implementation, and evaluation of AMS interventions from a One Health perspective. Using boundary object theory, we identified three key elements common to AMS in different settings in the human and animal health sectors—a sense of collective and temporal responsibility, flexibility in scale and scope, and contextual contingency. Based on these findings, we propose a definition for antimicrobial stewardship applicable to the human and animal health sectors, intended to facilitate intersectoral communication and cooperation. Further directions of this work could include the application of the definition to develop indicators for evaluating stewardship interventions and the extension of the definition to incorporate elements pertinent to plant and ecosystem health.

## Introduction

Antimicrobial stewardship (AMS) is a concept that relates to the use of antimicrobials in a way that preserves antimicrobial effectiveness while ensuring their ongoing availability for those who need them. It encompasses two different concepts relating to the appropriate use of antimicrobials—*conservation*, in determining *when and when not* to use antimicrobials, and *optimisation*, in including considerations of *how* to use antimicrobials appropriately. Antimicrobial stewardship therefore implies a comprehensive and contextualised approach to antimicrobial use (AMU), considering not only the quantities of antimicrobials used but moreover the ways in which and the reasons for which a range of different stakeholders (prescribers, patients, and others) use antimicrobials, and the context in which such decisions take place. The concept of AMS is becoming a core part of international efforts to address antimicrobial resistance (AMR)^1–4^, and many localised stewardship interventions designed and implemented over the last 20 years have aimed to reduce or optimise AMU in both human health^5,6^ and animal health sectors^7–9^. Because AMR has drivers and implications for human health, animal health, plant health, and the environment, it is increasingly being addressed using a One Health approach^10^. A One Health approach ‘recognises the health of humans, domestic and wild animals, plants, and the wider environment (including ecosystems) are closely linked and interdependent’ and notably includes stewardship in general (not only applied to AMU) as one of its key underlying principles^11^.

Antimicrobial stewardship has the potential for broad application in a range of different contexts—across different sectors (e.g., human, animal, and plant health), sociocultural environments (e.g., different countries or regions), as well as specific settings or infrastructures (e.g., hospital or outpatient settings, or different animal production systems, or ecosystems). This has led to a proliferation of context-specific definitions of AMS, such that the term is used interchangeably to refer to different concepts. For example, some definitions frame stewardship as a programme or intervention implemented and coordinated by a team in a hospital setting^12,13^, while other definitions focus on individual actors’ behaviours related to antimicrobial prescribing or use in a community setting^14,15^. Guidance from international organisations often frames AMS as a broad high-level strategy or approach to tackling AMR for implementation by a government or other authority, either at the national^16^ or international^3^ level. Although these different approaches to AMS are not mutually exclusive, they are suggestive of the variety in scope and scale at which AMS is applied at both a strategic and operational level and differences in where the principal responsibility for action lies. Antimicrobial stewardship implemented at a national, regional, community, or institutional level will necessarily imply different actions and different methods of application (including soft law such as policies or guidelines, hard law such as regulations, or development of local strategies). The importance granted to these actions and methods will vary depending on the perspective of different stakeholders (government, healthcare workers, veterinarians, farmers, or the community at large). This exemplifies the challenge of communicating the meaning of AMS to a wide range of stakeholders. This challenge is further exacerbated by the issue of translation, when languages other than English often have no direct equivalent for the word ‘stewardship’, referring rather to ‘good’ or ‘rational’ use^17^. While some overarching frameworks and definitions for AMS have been proposed^17–20^, these have largely been framed within the context of either human health or animal health, often to the exclusion of the other.

The absence of a universal definition of AMS across sectors presents key challenges. Firstly, it contributes to a lack of clarity, impeding communication on AMS and AMR both between different sectors and with the general public more broadly^21^. Given the need for a coordinated One Health effort to address AMR and create public awareness, this impact on communication is particularly problematic. Secondly, the lack of specificity in defining AMS makes it challenging to identify how stewardship can manifest in concrete actions in clinical practice or in the field. For example, many AMS definitions refer to ‘optimisation’ of use or clinical outcomes without providing a method for achieving this. For stakeholders in the field (doctors and nurses in primary care, pharmacists, veterinarians, farmers, and others) who may not use or attach the same meaning to the term ‘stewardship’, this can lead to a lack of clarity or confusion. Lastly, the absence of a clear AMS definition impedes the development of AMS indicators to measure progress. Measuring the success of stewardship programmes in hospital contexts in both uptake and impact often relies on structure and process indicators (presence of an AMS team, proportion of professionals who have received training) as well as outcome indicators (consumption/use, hospital re-admission rates, or mortality^22,23^). In veterinary contexts, AMU data from sales or prescription records are more commonly used^8,14^, although clinical and/or AMR data are sometimes included^9^. However, such indicators are dependent on many other factors than AMS, making attribution of causality difficult, and such data can be difficult to collect on a large scale. Alternative indicators to assess ‘appropriateness of use’ are increasingly being used in both the human and animal health sectors (such as documentation of indications, documentation of review or stop date, the proportion of surgical prophylaxis compliant with guidelines, use of diagnostic tools, and adherence to guidance)^24–26^. A clearer consensual understanding of what AMS is could facilitate the identification of and agreement on such indicators.

A coherent definition of stewardship is needed to address the issues elaborated above. The challenge is to identify a definition that is sufficiently flexible to be usable in different contexts by different communities across the One Health spectrum while remaining concrete enough to be of practical use for actors in the field. Our objectives are to:

- Identify how AMS is defined in different contexts and highlight commonalities.
- Develop an inclusive, intersectoral working definition of AMS that should be acceptable for the human and animal health sectors, with the potential for future application for plant and ecosystem health.

We have limited the scope of our work to human and animal health as the literature on AMS in plant health, ecosystem health, and the environment is relatively nascent and therefore scarce, although we recognise that these sectors are tightly linked to and influence human and animal health. We discuss definitions of both AMS and antibiotic stewardship (ABS), but we privilege the use of AMS as this is the broader term.

## Approach to developing a definition of antimicrobial stewardship

To develop a definition of stewardship applicable to different sectors and settings, we used boundary object theory, an approach first applied in science and technology studies to explain how scientific communities from different disciplines collaborate and cooperate without coming to consensus^27^. In doing so, we conceptualised AMS as a boundary object – an idea, concept, theory, or other object that facilitates collaboration by being ‘both adaptable to different viewpoints and robust enough to maintain identity across them’^27^. Boundary object theory has been applied to explain and theorise methods of cross-sectoral and transdisciplinary collaboration^28–31^, and thus has relevance for developing an AMS definition with a One Health approach. One of the hallmark features of a boundary object is ‘dynamic use’, referring to the common use of the object by scientists from different disciplines, who, in the process of collaboration, negotiate between a well-structured definition of the object for their local use, and a vaguer understanding of the object for collaborative work^32^. It is our aim to articulate this vague, shared understanding of AMS in the form of a definition that could be used by actors from different sectors without prohibiting or being incompatible with the more local definitions functional for their specific context.

Through an exploratory and iterative process, we reviewed the literature to identify commonalities across different definitions of stewardship, which could form the basis for a shared definition. This process began with reading on the concept of stewardship in general, then narrowed to stewardship in the context of public, animal, and ecosystem health (before excluding ecosystem health due to a lack of information). A list of definitions and explanations of stewardship and AMS was produced, from which key elements and characteristics common to these definitions were identified and subsequently refined into three shared elements. These elements became the basis for the proposed definition, which was developed through common discussion by the co-authors.

## Existing definitions and applications of antimicrobial stewardship

### A brief history of stewardship

The term ‘stewardship’ derives from Middle English in the 1400s and has since been applied in disciplines as diverse as environmental conservation and land sustainability^33–36^, agriculture^37^, organisational behaviour^38,39^, theology^40^, and accounting^41^. Modern definitions emphasise a sense of responsibility for a valuable and limited resource, often with a focus on moral obligation and ethical standards^42^. The concept of stewardship in the sense of guardianship or responsibility to preserve a precious resource has its counterpart in indigenous cultures, for example, the Māori concept of *kaitiakitanga*^43,44^. The first formal mention of ‘antimicrobial stewardship’ occurred in the 1990s^45^, in response to growing concern over rapidly increasing antibiotic resistance in hospitals, although interventions that would be recognised as AMS programmes today have been implemented since the 1970s^46^. During the 1990s, AMS became entrenched as a term referring to programmes or interventions designed to reduce AMU and thereby selection pressure for AMR within hospital settings. More recent recognition of AMU in community-based and animal health settings has broadened its scope. The need for AMS in animal health was highlighted within the context of human health in the 1990s^47^, with guidance on AMU in animals^48^ and principles of AMS for animal health being established later^49^. The term ‘antibiotic stewardship’ is also often mentioned in the aforementioned articles—usually to indicate considerations specific to antibiotics, but sometimes used interchangeably with AMS.

### Different sectoral definitions and uses of stewardship

Over time, context-specific definitions of ABS and AMS have evolved. These are largely functional to meet the needs of different sectors and their stakeholders. We provide a summary of how stewardship is applied in different settings for the human and animal health sectors, with a non-exhaustive list of representative definitions for each sector provided in Tables 1 and 2.

**Table 1:** Selected definitions of antimicrobial and antibiotic stewardship in the human health sector by setting

Setting	Definition	Source
Healthcare institution	‘Antimicrobial stewardship refers to coordinated interventions designed to improve and measure the appropriate use of antimicrobial agents by promoting the selection of the optimal antimicrobial drug regimen including dosing, duration of therapy, and route of administration. The major objectives of antimicrobial stewardship are to achieve the best clinical outcomes related to antimicrobial use while minimising toxicity and other adverse events, thereby limiting the selective pressure on bacterial populations that drives the emergence of antimicrobial-resistant strains. Antimicrobial stewardship may also reduce excessive costs attributable to suboptimal antimicrobial use.’	Policy Statement on AMS by SHEA, IDSA, and PIDS^13^
Outpatient / community	‘Antibiotic stewardship is the effort to measure antibiotic prescribing; to improve antibiotic prescribing by clinicians and use by patients so that antibiotics are only prescribed and used when needed; to minimise misdiagnoses or delayed diagnoses leading to underuse of antibiotics; and to ensure that the right drug, dose, and duration are selected when an antibiotic is needed.’	USA CDC guidance on outpatient AMS^74^
Global	‘A coherent set of actions which promote the responsible use of antimicrobials. This definition can be applied to actions at the individual level as well as the national and global level, and across human health, animal health and the environment.’	WHO guidance on AMS in LMICs^75^
	‘Antimicrobial stewardship is an organisational or healthcare system-wide approach to promoting and monitoring judicious use of antimicrobials to preserve their future effectiveness.’	EC, 2017^76^
	‘Antimicrobial stewardship is a coherent set of actions which promote using antimicrobials in ways that ensure sustainable access to effective therapy for all who need them.’	Dyar et al. ^17^

*AMS* antimicrobial stewardship, *EC* European Commission, *IDSA* Infectious Diseases Society of America, *LMICs* low- and middle-income countries, *PIDS* Paediatric Infectious Diseases Society, *SHEA* Society for Healthcare Epidemiology of America, *USA CDC* United States of America Centers for Disease Control and Prevention, *WHO* World Health Organisation.

**Table 2:** Selected definitions of antimicrobial and antibiotic stewardship in the animal health sector by setting

Setting	Definition	Source
Companion animals	‘The term “antimicrobial stewardship” is used to describe the multifaceted and dynamic approaches required to sustain the clinical efficacy of antimicrobials by optimising drug use, choice, dosing, duration, and route of administration, while minimising the emergence of resistance and other adverse effects. The word stewardship implies the obligation to preserve something of enormous value for future generations, and resonates in a way that “prudent use” or “judicious use” does not.’	Guardabassi & Prescott, 2015^20^
	‘Antimicrobial stewardship… refers to the actions veterinarians take individually and as a profession to preserve the effectiveness and availability of antimicrobial drugs through conscientious oversight and responsible medical decision-making, while safeguarding animal, public, and environmental health.’	AVMA^77^
	‘Antimicrobial stewardship encompasses all the individual and collective actions that medical professionals take to preserve the efficacy of antimicrobials. It is a one-health problem, affecting animals and humans.’	Cazer, 2023^78^
Companion and food production animals	‘Antimicrobial stewardship describes measures that can help mitigate the public health crisis and preserve the effectiveness of available antimicrobial agents.’	Lloyd & Page, 2018^19^
	‘Antimicrobial stewardship is the term increasingly used in medicine to describe the multifaceted approaches required to sustain the efficacy of antibiotics and minimise the emergence of resistance. The concept is still developing but includes a guiding 5 R set of principles of Responsibility, Reduction, Replacement, Refinement and Review.’	Weese, 2013^49^, Prescott, 2019^79^

*AVMA* American Veterinary Medical Association.

#### Human health

There are two distinct ways that stewardship is operationalised in human health. One set of definitions refers to AMS as a formal, coordinated intervention or programme commonly implemented in healthcare institutions, including long-term care facilities, and undertaken by multidisciplinary teams^13^. By contrast, the approach to stewardship may take on a different tenor in community-based settings, where it more commonly involves decision-making and AMU practices of individual practitioners—primary care doctors, pharmacists, midwives or dentists’ offices^50,51^. This illustrates that even within one single health sector, there are multiple different forms that stewardship can take.

The term ‘diagnostic stewardship’ has also been coined to describe the optimisation of diagnostic test use in infection management^52^. Critics point to the fact that, unlike antibiotics, diagnostic tests are more commonly under-utilised and do not need ‘preserving’ since they do not lose efficacy when used, and that use of this term potentially downplays the element of collective responsibility, which is part of AMS. Others have concerns that the term suggests a separation of the diagnostic process from AMS^17,53^.

#### Animal health

Stewardship in animal health settings may be implemented in a variety of ways, in part because the animal health sector represents a heterogeneous group of subsectors with different AMU practices^54^. There are notable differences between AMU practices in companion animal species and food animal species, and strategies to preserve antimicrobial effectiveness will differ depending on whether the health of animals is managed at an individual or population level—for example, in the latter case, stewardship interventions can be made throughout the lifespan of a group of animals. There is also variety in the range of reasons for which antimicrobials are used. ‘Veterinary medical use’ of antimicrobials (as defined by the World Organisation for Animal Health (WOAH)) is considered to include treatment, prophylaxis, and metaphylaxis^55^. Antimicrobials may also be used for non-veterinary medical use, in particular growth promotion, although increasingly, countries are implementing regulations to ban or phase out this practice^56^.

Veterinarians prescribing antimicrobials must also consider the potential implications of AMU for both animal and human health—AMU decisions are often driven by concerns of resistance development in humans rather than in animals themselves. For example, restrictions have been placed on the use of specific, critically important antibiotics in animals because of their importance to humans^57^, such as fluoroquinolones in poultry^58^. Veterinarians also must contend with welfare implications, and economic and emotional consequences of decisions to use or not use antimicrobials, and in the case of food production animals, considerations of food safety and food security.

#### Global/international organisations

It appears that there is no global consensus on an AMS definition. This is suggested by the absence of a definition formally endorsed by any of the Quadripartite organisations that work on AMR as a One Health topic—the World Health Organisation (WHO), WOAH, the Food and Agriculture Organisation (FAO), and the United Nations Environmental Programme (UNEP)—or by Codex Alimentarius. While there is a definition for AMS in a WHO guidance document (Table 1), we could not find evidence of a definition formally endorsed by the World Health Assembly or by the corresponding decision-making bodies at WOAH and FAO. The closest definition provided by WOAH is the definition of “responsible and prudent use” from Chapter 6.10 of WOAH’s *Terrestrial Animal Health Code*^59^, as WOAH does not currently use the term ‘AMS’. No definitions could be identified for FAO, who do not mention ’stewardship’ in their *Antimicrobial Resistance Terms*^60^, or Codex Alimentarius in their standards on AMR^61^. This is perhaps indicative of the preference for other terms such as ‘responsible’, ‘prudent’, or ‘judicious’ use. Having a unified definition endorsed by the Quadripartite could facilitate building a shared understanding both within and between the different sectors.

### Identification of what is common to most definitions of stewardship

The use of boundary object theory allowed us to identify three elements of AMS that are common to the different definitions above and the stewardship literature more generally—a sense of moral (collective and temporal) responsibility, flexibility in scope and scale, and contextual contingency.

#### Stewardship as a moral (collective and temporal) responsibility

The notion of responsibility and accountability to others is an overarching theme in stewardship dating back to the 1400s. Over time, the entity that stewards are accountable to has shifted from an individual authority (e.g., a landowner) to a broader collective (e.g., society as a whole or future generations). This change indicates the shift that has taken place in definitions of stewardship, from being predominantly about *management* to incorporating broader questions of *responsibility*, imbuing the term ‘stewardship’ with a moral and ethical dimension. As the use of antimicrobials impacts society as a collective, this responsibility is framed as an accountability that extends in two directions: *collectively*, from the individual prescriber or patient being treated to society as a collective (intragenerational reciprocity), and *temporally*, from the present beneficiaries of AMU to future generations who will experience the consequences of AMU decisions made in the present (intergenerational reciprocity). Most definitions reflect this conflict, suggesting that AMS is about a balance between competing needs when deciding whether to use antimicrobials at all (see Table 3) and describing the need to use antimicrobials in a responsible manner—commonly also referred to as ‘optimal’, ‘judicious’, ‘prudent’, or ‘appropriate’ use (see Table 4).

**Table 3:** Text excerpts on stewardship as being about a ‘balance of needs’

Context	Text excerpt
Stewardship in general	‘…stewardship theory implies the organisational goal of sustainability—that is, meeting the needs of the present without compromising the ability of future generations to meet their own needs.’^38^
Stewardship in general	‘Stewardship … takes full and balanced account of the interests of society, future generations, and other species, as well as of private needs, and accepts significant answerability to society.’^36^
Human health	‘The major objectives of antimicrobial stewardship are to achieve best clinical outcomes related to antimicrobial use while minimising toxicity and other adverse events…’^13^
Human health	‘…antimicrobial stewardship is about using antimicrobials responsibly, which involves promoting actions that balance both the individual’s need for appropriate treatment and the longer-term societal need for sustained access to effective therapy.’^17^
Animal health	‘…veterinarians will be forced to prioritise among these obligations even though they have no desire to act in a manner that is harmful to the public, their patients, or their clients.’^48^
Animal health	‘The prescribing veterinarian accepts responsibility for the decision to use an antimicrobial agent and recognises that such use can have adverse consequences beyond the recipient.’^49^

**Table 4:** Text excerpts on stewardship consisting of ‘optimal’, ‘judicious’, ‘responsible’, ‘prudent’, or ‘appropriate’ use

Context	Text excerpt
Human health	‘…selection of the optimal antimicrobial drug regimen including dosing, duration of therapy, and route of administration.’^13^
Human health	‘…ensure that the right drug, dose, and duration are selected when an antibiotic is needed.’^74^
Human health	‘…actions which promote the responsible use of antimicrobials.’^75^
Human health	‘…approach to promoting and monitoring judicious use of antimicrobials…’^76^
Animal health	‘…optimising drug use, choice, dosing, duration, and route of administration…’^20^
Animal health	‘Ensure that each patient receives the most appropriate treatment: the right drug, at the right time, at the right dose for the right duration by the right route of delivery (5 rights).’^19^

#### Flexibility in scope and scale—all actors at all scales can contribute to stewardship

Many AMS definitions refer to the range of different actors implicated in stewardship and the variety of ways in which they can contribute. Some definitions relate to the specific roles of individuals, and others to a system-wide approach that should be enacted throughout different levels of an organisation or even involving many different institutions (Table 5). This aspect also highlights the multidisciplinary and intersectoral aspect of stewardship by suggesting that the involvement of individuals or institutions from different sectors or disciplines is important to help drive stewardship efforts.

**Table 5:** Text excerpts on the need for stewardship to be broad in scope and scale

Context	Text excerpt
Stewardship in general	‘…stewardship behaviours can be enacted across all levels of the organisation.’^38^
Stewardship in general	‘Stewardship actions can occur at different scales, can address issues that are of greater or lesser complexity, and are taken by different individuals or groups of actors because of their motivations and available capacities.’^34^
Human health	‘This definition can be applied from individual-level actions to global level actions, and across human health, animal health and the environment.’^17^
Animal health	‘Everyone associated with antibiotic use, whether government regulators, individual veterinarians or animal owners, needs to be involved in a stewardship approach.’^49^
Animal health	‘Stewardship thus links, for example, front-line veterinary practitioners with laboratory diagnosticians, owners, drug regulators, and pharmaceutical companies.’^20^

Antimicrobial stewardship actions (the specific activities, decisions, and behaviours taken by individual actors) and interventions (coordinated policies or programmes implemented by individuals or organisations) can vary in scope and scale:

1. *Scope* is defined as the boundaries placed on where stewardship actions/interventions are enacted. This includes the types of practices covered. For example, the scope may be limited to prescribing or use practices, may include the development of guidelines, monitoring, or education campaigns, or in some cases, be closely linked to preventive measures to reduce the need for antimicrobials in the first place, a major feature of livestock AMS.
2. *Scale* is defined as the level at which stewardship is enacted, be it geographic (e.g., global, national, regional), sectoral (e.g., human, animal, plant, vs. One Health), for a given target population (e.g., community-based vs. institutional), or level of involvement within a community/institution (e.g., individual prescribers vs. a hospital management board for human health, or animal owners vs. veterinarians vs. veterinary authority for animal health).

The scope and scale of AMS can be variable. For example, there is debate over whether preventive measures—infection prevention and control (IPC) measures targeting both healthcare-associated and community-acquired infections in humans, safe and sufficient water, sanitation and hygiene (WASH) in humans, vaccination (in humans and animals), and biosecurity on farms, among others—are included within the scope of stewardship. In human health, IPC measures are often considered to be separate from AMS, in part because they contribute to the broader goal of infection prevention, not only for the purpose of reducing the need for antibiotics and transmission of antibiotic resistance. In animal health, decisions to implement preventive measures such as vaccination or biosecurity are often managed by the same individuals responsible for overseeing AMU (i.e., veterinarians) and may be considered together, particularly within a herd or flock health management plan in food production species. In both human and animal health, although actions undertaken for infection prevention are separate from those related to AMU practices, they exist along a continuum of different AMS and AMR actions, all of which contribute to the broader goal of preserving antimicrobial efficacy.

By defining the scope and scale of AMS, the actors required and the setting in which they act can be more easily identified. The roles or actions associated with AMS might vary for different individuals at different scales, but all are considered essential to implementation.

#### Contextual contingency—the importance of context

Most definitions or explanations of stewardship emphasise that implementing stewardship interventions or actions is inherently context-based. What ‘good’ stewardship means as a concept will not change. However, the stewardship actions to be implemented will vary depending on the context. Context includes a variety of barriers and facilitators, including social, cultural, political, and economic factors, as well as the characteristics and influence of individual actors, their interrelationships, the institutional location, and the surrounding infrastructure^62,63^. It is also linked to notions of capacity—suggesting that ‘good’ stewardship implies taking the most appropriate course of action under a set of given circumstances and given currently available resources. These considerations are pertinent for resource-limited settings, such as low- and middle-income countries (LMICs) or rural and remote geographic areas, where limited access to medical and veterinary services and/or good-quality antibiotics restrict the capacity for compliance with guidelines. However, they are also relevant for high-income countries and in specialised medical and veterinary settings, where the availability (or lack thereof) of resources will influence the capacity of individuals and institutions to implement stewardship actions (for example, lack of protected time for staff to perform stewardship functions in hospitals, or prohibitive costs of culture and susceptibility testing in veterinary practice). Some excerpts acknowledging the importance of context for AMS are provided in Table 6.

**Table 6:** Text excerpts on the importance of context for stewardship

Sector	Text excerpt
Stewardship in general (environmental)	‘…the broader social–ecological context determines which stewardship actions will be socially, culturally or politically feasible, appropriate or effective. In different cultural contexts, the types of stewardship actions that will be deemed appropriate will differ.’^34^
Human health	‘both the coherent set of actions and their objective (using antimicrobials responsibly) are inherently context-specific and will vary depending on who is doing them: the evidence base of interventions evolves over time, what works in one place may not work or be coherent in others, and what is considered responsible use at one time-point may not be considered responsible at another’^17^
Animal health	**‘**An antimicrobial stewardship programme in a small clinic may look quite different from the programme for a referral hospital, but every veterinary practice can contribute to the reduction of antimicrobial resistance by developing, instituting, and following an antimicrobial stewardship program that works for its particular setting.’^80^

## A definition of antibacterial stewardship with a One Health perspective

Drawing on the elements that we identified above as common to different definitions of AMS and ABS across the human and animal health sectors—collective and temporal responsibility, flexibility in scope and scale, and contextual contingency—we propose the following definition for antimicrobial stewardship (Box 1):

This definition is intended to facilitate communication and understanding of AMS across different sectors and settings. It suggests consensus on the broader concept of what stewardship represents but recognises that the specific actions required to operationalise the stewardship principles in this definition will be contingent on the context. It eschews the use of words which imply some degree of judgement (appropriate, rational, prudent, responsible, judicious) or are difficult to translate into languages other than English and focuses instead on what stewardship aims to achieve—a balance between the availability of antimicrobials for the present, and the preservation of antimicrobial effectiveness for the future.

Box 1 **Antimicrobial stewardship**: A concept relevant to and applicable by all (individuals, communities, and institutions) *[scope and scale]*, aiming at using and prescribing antimicrobials in humans and animals in a way that ensures the availability of antimicrobials for individuals in the present day, as well as preserving antimicrobial effectiveness for current and future populations *[collective and temporal responsibility]*. The operationalisation of stewardship includes considerations of whether antimicrobials should be used, the ways in which antimicrobials are used, as well as the broader context within which these decisions are made *[contextual contingency]*.

## Discussion and perspectives

Antimicrobial stewardship is an essential concept for addressing the challenge of AMR and the potential loss of antimicrobial effectiveness, but it is at risk of losing significance due to a lack of clarity about what it is understood to mean. Antimicrobial use practices vary by context^64,65^, so it is therefore not so much inconsistent as perfectly logical that different operational definitions of stewardship would be used in different contexts. Nonetheless, for the purpose of communication and intersectoral work, it is important to have some shared understanding of the concept of ‘antimicrobial stewardship’, and for this reason, we sought to establish a shared conceptual definition. We developed a definition of AMS using boundary object theory.

Boundary object theory has been applied to other concepts with transdisciplinary and intersectoral applications, including resilience^66,67^, ecological indicators^68^, ecosystem services^31^, and landscape stewardship^35^. However, when stewardship was considered, disciplines relating to AMS (including medicine, veterinary science, and microbiology) were explicitly excluded^35^. We considered that AMS functions as a boundary object because it possesses the characteristic of being ‘weakly structured in common use, and … strongly structured in individual-site use’^27^. Definitions of AMS may appear vague when viewed collectively. However, the operational definitions used by specific communities within the human and animal health sectors were often functional, meaningful, and well-structured for that community. The use of boundary object theory allowed us to identify the elements common to existing AMS definitions across different settings and sectors and to shape these into a common definition. The proposal of a shared definition is not meant to prevent the use of more localised definitions specific for given contexts across One Health sectors, but rather, to co-exist alongside them and function as a communicative tool for intersectoral collaboration—allowing actors to agree in essence on what they may disagree on in practice. ‘Boundary objects do not claim to represent universal, transcendent truth; they are pragmatic constructions that do the job required’^69^—in our case, the job required was to facilitate communication around a common object. This approach has the potential for future application to facilitate communication on other similarly challenging topics in One Health.

The importance of context was one of the key elements of our definition. Contextualising the design of AMS interventions is important due to the barriers and enablers that can be encountered. It has been shown that AMR is associated with a range of contextual factors, such as poor infrastructure, poor governance, and low healthcare expenditure, which can both influence AMU behaviours directly and contribute to AMR dissemination^70^. This has two important implications for AMS interventions. Firstly, the menu of stewardship interventions that may be effective (based on the literature) will need to be adapted to the specific context where they are being implemented. For example, access to antimicrobials and diagnostics varies considerably – in the global south, lack of access to doctors, veterinarians, and diagnostic services (e.g., laboratory support and point-of-care tests) often means that populations are dependent on the informal sector to obtain antimicrobials^54,71^. Stewardship in such contexts will likely take a different form than in areas where access to healthcare is better. It is for this reason that contextual factors (including cultural factors) should be described during the design of stewardship interventions and considered throughout their implementation. Secondly, stewardship can only do so much. Even where AMS interventions are adapted to the context, it will be difficult to bring about meaningful change without longer-term efforts to address more structural, intransigent problems, which also contribute to AMR.

The scope of our definition also bears discussing. Our definition is explicitly for *antimicrobial* stewardship. It is important to acknowledge that much of the literature drawn on for this definition also referred to *antibiotic* stewardship. We acknowledge that stewardship considerations at a practical level may be different for viruses, parasites, protozoa, or fungi than they are for bacteria—however, as our definition is framed at the level of general principles, we anticipate that these principles should have applicability or adaptability for stewardship for a range of different pathogens. Nonetheless, we think it is important to highlight that there is a broader trend of the interchangeable use of ‘antimicrobial’ with ‘antibiotic’ without additional explanation or justification, such that AMS is often implicitly understood to refer only to ABS. Such laxity of language contributes to confusion about what AMS really means and can have important implications in the context of guidelines or regulations where specific measures or approaches in the clinic or field will need to be tailored for different kinds of pathogens. It also limits the applicability of AMS—if AMS is described in a way that only has relevance for bacteria, considerations for antifungal and antiparasitic stewardship are at risk of being lost or at least deprioritised. So too, are plant and environmental health, as the sectors for which these types of resistance are of particular importance^72^. The prioritisation of antibacterial/antibiotic resistance within AMS to the detriment of resistance to other types of pathogens may be one of the reasons for the infrequent use of the term ‘antimicrobial stewardship’ in the context of plant and ecosystem health.

Partly as a consequence of this, our definition focused on human and animal health, excluding plant and ecosystem health. The term ‘antimicrobial stewardship’ was uncommon in the literature on AMU/AMR in plant and ecosystem health, which often focuses on AMR in the environment through the lens of the potential impact on human and animal health^73^. Furthermore, although we found an abundance of varying but precise definitions for AMS in human and animal health (in the terms of boundary object theory, definitions that were ‘strongly structured in individual-site use’^27^), we found no such well-structured definitions of AMS in the context of plant or ecosystem health to inform our shared definition. However, the stewardship principles identified in our definition are sufficiently broad that they should apply and can likely be adapted and implemented. As global efforts to work across the One Health spectrum increasingly grow more inclusive (for example, the recent shift from the Tripartite collaboration on AMR to the Quadripartite by the inclusion of UNEP), the concept of AMS may become more widely used in plant and ecosystem health contexts. In the absence of greater information on what AMS might look like in these sectors, it is hoped that this definition, developed for use in human and animal health, serves as a first step towards the development of a definition equally applicable across all One Health sectors.

Our proposed definition of AMS is relevant to human and animal health and each sector’s varying contexts. We believe that the proposed definition will facilitate intersectoral communication and cooperation by providing a coherent explanation of AMS relevant to the different sectors implicated in One Health AMS interventions and by encouraging more explicit consideration of what AMS means. We urge the scientific community to adopt a common, inclusive definition for AMS, and when applying AMS in a specific context, to articulate clearly what is intended by the term ‘AMS’. The adoption of a shared definition or a minimum consensus on the scope of AMS by international human and animal health organisations would greatly contribute to this goal, and we hope that our definition may serve as a prompt for the prioritising definition of this term within the relevant organisations’ work plans. The next step to make this definition of AMS more concrete would be the articulation of indicators, which may serve to make stewardship more meaningful for actors in the field who are the target of AMS interventions.
